# Prognostic Value of Deep Learning‐Extracted Tumor‐Infiltrating Lymphocytes in Esophageal Cancer: A Multicenter Retrospective Cohort Study

**DOI:** 10.1002/cam4.71054

**Published:** 2025-07-17

**Authors:** Peishen Li, Shujie Huang, Haijie Xu, Zijie Li, Sichao Wang, Zhen Gao, Yuejiao Dong, Zhuofeng Chen, Guibin Qiao, Hansheng Wu, Liangli Hong

**Affiliations:** ^1^ Department of Thoracic Surgery The First Affiliated Hospital of Shantou University Medical College Shantou China; ^2^ Department of Thoracic Surgery Guangdong Provincial People's Hospital (Guangdong Academy of Medical Sciences), Southern Medical University Guangzhou China; ^3^ Shantou University Medical College Shantou China; ^4^ Department of Pathology The First Affiliated Hospital of Shantou University Medical College Shantou China

**Keywords:** deep learning, esophageal squamous cell carcinoma, prognostic factors, tumor‐infiltrating lymphocytes

## Abstract

**Background:**

Tumor‐infiltrating lymphocytes (TILs) have been proven to be important prognostic factors for various tumors. However, their prognostic significance within the context of esophageal squamous cell carcinoma (ESCC) remains inadequately explored. This study aims to assess the prognostic potential of TILs in ESCC using deep learning (DL) methods.

**Materials and Methods:**

We retrospectively enrolled 626 pathologically confirmed ESCC patients from two research centers. Their digital whole‐slide imaging (WSI) and corresponding clinical information were collected. Subsequently, the DL method was employed to identify the tumor margin and TILs within the WSI. Tissue was divided into intratumor, peritumoral, and stromal regions based on their distance from the tumor margin. TILs were counted in each region. Optimal cut‐off values of TILs were determined using the X‐tile software. To mitigate selection bias and intergroup heterogeneity, a propensity score matching (PSM) analysis was employed. Survival analysis was performed using Kaplan–Meier curves and the log‐rank test. The Cox proportional hazards regression model was used to identify independent prognostic factors.

**Results:**

We classified patients based on the cell counts and cut‐off values of intratumor‐infiltrating lymphocytes (I‐TILs) and peritumoral infiltrating lymphocytes (P‐TILs). Patients with high I‐TILs and P‐TILs were defined as those whose counts of both I‐TILs and P‐TILs exceeded the determined cutoff value. Patients with high I‐TILs and P‐TILs showed significantly better overall survival (OS, *p* = 0.0092) and recurrence‐free survival (RFS, *p* = 0.0088) than patients with low I‐TILs and P‐TILs after PSM. Multivariable Cox proportional hazards regression further supported this conclusion and recognized I‐TILs and P‐TILs as independent prognostic factors (*p* = 0.0136, hazard ratio = 0.63 for OS; *p* = 0.0098, hazard ratio = 0.63 for RFS).

**Conclusion:**

In the present study, we identified the quantitative distribution of TILs in ESCC patients with the help of the DL method. We established that I‐TILs and P‐TILs serve as independent prognostic factors for these patients. Further studies should focus on the lymphocyte subgroups and make better use of the spatial information to improve the predictive efficacy of TILs.

## 1 Introduction

Esophageal cancer is one of the most aggressive cancers and stands as the sixth leading cause of cancer‐related deaths worldwide [1, 2]. In Asia, 90% of the esophageal cancer cases are esophageal squamous cell carcinoma (ESCC) [3, 4, 5]. Despite the advances in multidisciplinary treatment, the prognosis of ESCC patients remains dismal. There exists an urgent need for the development of reliable prognostic predictors that can facilitate personalized treatment strategies and risk assessment.

Tumor‐infiltrating lymphocytes (TILs) refer to the lymphocyte population that is within tumor nests or in direct contact with tumor cells [6]. Considering the potential antitumor function of TILs, various features of TILs have been established as important prognostic and predictive factors in patients with various tumors [7, 8, 9, 10, 11, 12, 13]. Patients with a higher proportion of TILs have better survival outcomes in both early‐stage and advanced‐stage ESCC [13, 14]. Conversely, PD‐1^+^ and TIM‐3^+^ TILs, which represent immune exhaustion, are associated with poorer survival outcomes in various cancers [15]. Besides the proportion, the density of TILs also holds a significant prognostic value for patients. Through a retrospective analysis of 198 patients with ESCC who had undergone surgical resection, Chu et al. identified a significant association between a high density of intratumor CD103^+^ TILs and prolonged overall survival (OS) and disease‐free survival (DFS) [16]. In terms of spatial characteristics, it has been demonstrated that generalized TILs infiltrating the entire tumor, as opposed to solely intra‐tumor or peritumoral TILs, are correlated with favorable OS and DFS across various cancer types [12]. Despite substantial research efforts directed toward TILs, there exists a noticeable paucity of dedicated investigation into ESCC in comparison to other malignancies. Moreover, studies elucidating the spatial distribution of TILs within the context of ESCC are particularly scarce. This knowledge gap has resulted in an incomplete understanding of the prognostic importance of TILs for ESCC patients. Thus, there is a pressing need for more research in this field.

Digital whole‐slide imaging (WSI) has provided new possibilities for the pathological data analysis of tumors [17]. In recent years, the application of deep learning (DL) for pathological image analysis has seen a significant rise. Particularly in spatial features analysis, DL can extract more spatial information and make many complex analysis methods possible [18, 19]. However, there is a dearth of relevant research conducted in ESCC.

In the present study, we use a DL method to explore the calculation and the spatial distribution of TILs within hematoxylin and eosin (H&E) stained ESCC samples. We aimed to identify TILs‐related prognostic factors for ESCC who underwent esophagectomy.

## 2 Materials and Methods

### 2.1 Study Cohort

This study was authorized by the ethics committee of the Guangdong Provincial People's Hospital (GDPH) (No. GDREC2020142H) and the First Affiliated Hospital of Shantou University Medical College (SUMCFH) (No. 2020‐094). Written informed consent was obtained from all the patients. This study was conducted under the Declaration of Helsinki (as revised in 2013).

**FIGURE 1:**
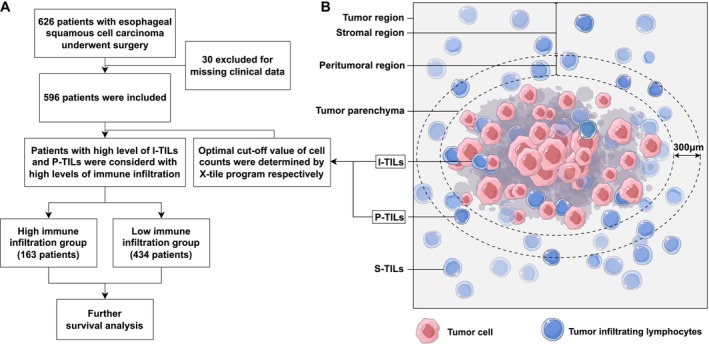
Study flowchart and tumor region division. (A) Study flowchart for the present study. (B) Division of tumor regions and lymphocyte nomenclature in different regions. The peritumoral region was defined as the area within a distance of up to 300 μm from the tumor margin. I‐TILs, intra‐tumor infiltrating lymphocytes; P‐TILs, peritumoral infiltrating lymphocytes; S‐TILs, stomal tumor infiltrating lymphocytes.

Pathologically confirmed ESCC patients from GDPH and SUMCFH who received surgical resection between 2010 and 2019 were included in this study. For each patient, the following clinical information was collected from the hospital information system: age (years), sex, tumor location, pathologic stage, histological grade, resection margin, lymphovascular invasion (LVI). Follow‐up information was obtained by telephone and outpatient service. Patients were excluded if any clinical data required for the study were missing (Figure 1A). Postoperative tumor tissues of patients were fixed with formalin, embedded in paraffin, sectioned into 4 mm, and followed by H&E staining. WSIs were imaged at a 40× magnification (Aperio, ScanScope AT2, Leica), with a resolution of 0.25 μm.

### 2.2 Model Architecture of Nuclei Segmentation and Classification

**FIGURE 2:**
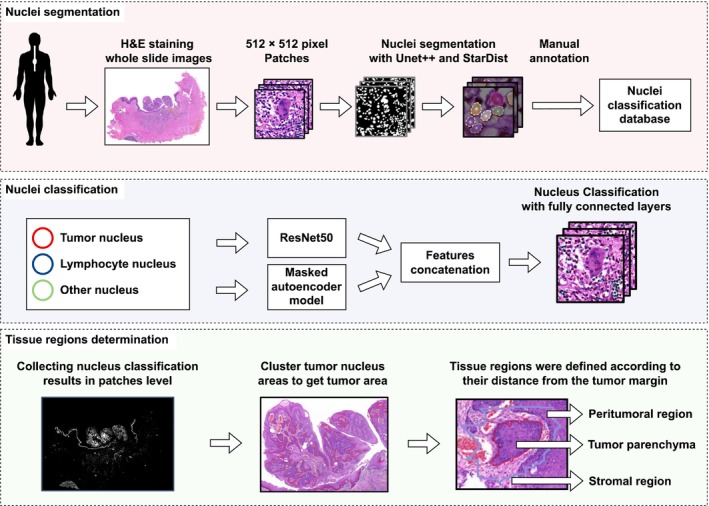
Workflow for TILs detection and tissue regions determination. Red: to establish a nuclei classification database, H&E stained whole slide images were broken into patches and cell and nuclei were segmented with U‐Net++ and StarDist. All the nuclei were manually annotated by pathologists. Blue: We fused the features extracted from ResNet50 and the Masked auto‐encoder model and used fully connected layers to achieve the classification of cell nuclei. Green: The tumor region was identified through clustering of the tumor nucleus region and the tissue regions were delineated according to their distance from the tumor margin. TILs, tumor infiltrating lymphocytes.

In this study, we employed a DL approach that was originally established in our previous work. A concise workflow for the model foundation is presented in Figure 2. H&E stained ESCC WSI were divided into multiple square patches with a resolution of 512 × 512 pixels. U‐Net++ demonstrates superior performance in semantic segmentation tasks through its nested skip connections and adaptive feature aggregation mechanisms, particularly excelling in high‐precision applications such as medical image analysis. While alternative architectures including DeepLabv3+, SegNet, and PSPNet exhibit context‐specific advantages, U‐Net++ and its variants generally achieve enhanced segmentation accuracy and overall performance metrics [20, 21]. The StarDist model, a deep learning‐based framework for nuclear segmentation, has garnered significant attention in biomedical image processing. Compared to conventional methods such as Mask R‐CNN and U‐Net, StarDist exhibits unique strengths in nuclear instance segmentation. Instead of directly predicting pixel‐level segmentation masks, StarDist learns to parameterize star‐convex polygons for each nucleus [22]. This representation demonstrates robustness to imaging noise and effectively accommodates nuclear morphological variability. Unlike traditional approaches that perform pixel‐wise classification, StarDist defines nuclear boundaries by predicting polygon vertices, thereby enhancing segmentation efficiency and accuracy, particularly in densely packed nuclear regions [23, 24]. Consequently, the U‐Net++ network was employed for the semantic segmentation of cells [21]. Subsequently, H&E‐stained nuclei were segmented using an already trained model “StarDist” [25]. Upon acquiring all the segmented cell nuclei, two experienced pathologists were invited to conduct an independent evaluation of the image quality and annotate the types of cell nuclei. They classified the cell nuclei as tumor nuclei, lymphocyte nuclei, or other nuclei based on their expertise. Under H&E staining, conspicuous differences can be observed in the nuclei between esophageal cancer tumor cells and lymphocytes. The nuclei of esophageal cancer tumor cells exhibit remarkable characteristics, such as attaining a substantial size, demonstrating high heterogeneity, and possessing irregular shapes. Moreover, the distribution of chromatin within these tumor cells is nonuniform, with manifestations of marginal aggregation and blocky aggregation. Conversely, the nuclei of lymphocytes are relatively diminutive, predominantly presenting in regular round or oval configurations. The chromatin therein exists in the form of fine granules and is evenly distributed throughout the nuclei. These differences are of great assistance to pathologists in making identifications and serve as a solid basis for diagnosis and evaluation.

ResNet50, a classic convolutional neural network, and a self‐supervised masked autoencoder (MAE) model [26, 27]. Sharma et al. have discovered that ResNet50 is competent in discriminating among neutrophils, eosinophils, basophils, monocytes, lymphocytes, and other white blood cell subtypes, which has a 95% accuracy rate in balanced precision, recall, and f1‐scores for categorizing white blood cells [28]. Sornapudi et al. have contrasted a series of CNN models and have ascertained that ResNet50 and VGG19 exhibit the optimal performance in cell classification [29]. For nuclei classification, we trained ResNet50. By fusing features extracted from ResNet50 and MAE, our DL approach achieved notable results in the nuclear segmentation task, with a mean Intersection over Union of 0.9369 and a mean Dice coefficient of 0.9209. The areas under the curve for lymphocyte, tumor cell, and stromal cell classifications stood at 0.92, 0.90, and 0.86, respectively.

### 2.3 Determination of Tissue Region

Using the DL model, we obtained the categories and locations of all nuclei in the WSI. To accurately identify tumor and nontumor regions, we conducted a series of operations, including morphological operations, tree/graph/geometry algorithms, and regional clustering algorithms on the contour of nuclei. Furthermore, we defined a peritumoral region located 300 μm beyond the tumor boundary. Nontumor regions located more than 300 μm outside the tumor boundary were defined as stromal regions (Figures 1B and 2) [18]. Detailed spatial partitioning allows for effective utilization of the spatial information of TILs. The code used in this study is openly accessible on GitHub (https://github.com/lpatience/Cancer‐Medicine).

### 2.4 TILs Feature Extraction

The characteristics of TILs were categorized into two primary sections: quantitative features and spatial features. Regarding quantitative features, each TIL was identified by the DL model, and its quantities were counted. For the purpose of reducing the deviation resulting from unequal regions, prior to the implementation of HE staining, blank slides of identical area were selected for each patient. As for spatial features, TILs were further divided into intratumor infiltrating lymphocytes (I‐TILs), peritumoral infiltrating lymphocytes (P‐TILs), and stomal tumor‐infiltrating lymphocytes (S‐TILs) based on the spatial relationship between tumor and TILs. Additionally, the TIL count was recorded for each specific region.

### 2.5 Statistical Analysis

Data were presented as mean ± standard deviation or median (interquartile range), depending on its distribution. Categorical variables were presented as frequencies and percentages. We performed comparisons of continuous variables using the Student's *t*‐test or the Wilcoxon test, whereas the chi‐squared test or Fisher's exact test was used for the comparison of categorical variables. Cutoff values were derived using the X‐tile program version 3.6.1 (Yale University School of Medicine, New Haven, CT, USA). OS was characterized as the duration of surgery until either death or the last follow‐up. Recurrence‐free survival (RFS) was defined as the period from surgery to clinically detectable recurrence. Survival analysis was performed using Kaplan–Meier curves and the log‐rank test. Propensity score matching (PSM) analysis was adopted to further reduce the selection bias and between‐group heterogeneity. We implemented Cox proportional hazards regression models to identify potential prognostic factors. All tests were two‐sided, with a *p* value < 0.05 indicating statistical significance. All statistical analyses were performed using R version 4.2.1 (R‐project, Institute for Statistics and Mathematics, Vienna, Austria) [30].

## 3 Results

### 3.1 Demographic and Clinical Characteristics

In our research, 626 WSIs were incorporated for TILs detection, with 311 from GDPH and 315 from SUMCFH. However, we excluded 30 samples during statistical analysis because of missing essential clinical data. Table 1 presents detailed demographic and clinical characteristics. Most of the enrolled cases were diagnosed as stage II and stage III diseases, accounting for 80.2% of the total cohort. The median follow‐up period for patients was 42.4 (IQR: 23.6–63.3) months in the GDPH cohort and 44.7 (IQR: 29.0–71.1) months in the SUMCFH cohort.

**TABLE 1:** Demographic and clinical characteristics of patients.

Variables	Total	Before PSM			After PSM		
		I‐TILs and P‐TILs		*p*	I‐TILs and P‐TILs		*p*
		Low	High		Low	High	
Total	596	433	163		163	163	
Age (median [IQR])	61 [55, 66]	61 [56, 66]	60 [55, 66]	0.424	60 [54, 65]	60 [55, 66]	0.472
Sex				0.432			1.000
Female	135 (22.7)	94 (21.7)	41 (25.2)		41 (25.2)	41 (25.2)	
Male	461 (77.3)	339 (78.3)	122 (74.8)		122 (74.8)	122 (74.8)	
Tumor location				0.232			0.983
Distal	149 (25.0)	269 (62.1)	35 (21.5)		36 (22.1)	35 (21.5)	
Mid	371 (62.2)	50 (11.5)	102 (62.6)		102 (62.6)	102 (62.6)	
Proximal	76 (12.8)	68 (15.7)	26 (16.0)		25 (15.3)	26 (16.0)	
Pathologic T stage				0.494			0.310
T1	98 (16.4)	68 (15.7)	30 (18.4)		29 (17.8)	30 (18.4)	
T2	148 (24.8)	114 (26.3)	34 (20.9)		48 (29.4)	34 (20.9)	
T3	344 (57.7)	247 (57.0)	97 (59.15)		85 (52.1)	97 (59.15)	
T4	6 (1.0)	4 (0.9)	2 (1.2)		1 (0.6)	2 (1.2)	
Pathologic N stage				0.280			0.430
N0	369 (61.9)	260 (60.0)	109 (66.9)		108 (66.3)	109 (66.9)	
N1	130 (21.8)	102 (23.6)	28 (17.2)		37 (22.7)	28 (17.2)	
N2	68 (11.4)	48 (11.1)	20 (12.3)		14 (8.6)	20 (12.3)	
N3	29 (5.0)	23 (5.3)	6 (3.7)		4 (2.5)	6 (3.7)	
Pathologic TNM stage				0.543			0.957
I	89 (14.9)	60 (13.9)	29 (17.8)		29 (17.8)	29 (17.8)	
II	293 (49.2)	212 (49.0)	81 (49.7)		82 (50.3)	81 (49.7)	
III	185 (31.0)	138 (31.9)	47 (28.8)		48 (29.4)	47 (28.8)	
IV	29 (4.9)	23 (5.3)	6 (3.7)		4 (2.5)	6 (3.7)	
Histological grade				0.811			0.807
G1	69 (11.6)	52 (12.0)	17 (10.4)		23 (14.1)	17 (10.4)	
G2	416 (69.8)	303 (70.0)	113 (69.3)		108 (66.3)	113 (69.3)	
G3	105 (17.6)	73 (16.9)	32 (19.6)		31 (19.0)	32 (19.6)	
Gx	6 (1.0)	5 (1.2)	1 (0.6)		1 (0.6)	1 (0.6)	
Resection margin				0.612			1.000
R0	576 (96.6)	417 (96.3)	159 (97.5)		159 (97.5)	159 (97.5)	
R1	20 (3.4)	16 (3.7)	4 (2.5)		4 (2.5)	4 (2.5)	
Lymphovascular invasion				0.881			0.504
Negative	533 (89.4)	388 (89.6)	145 (89.0)		140 (85.9)	145 (89.0)	
Positive	63 (10.6)	45 (10.4)	18 (11.0)		23 (14.1)	18 (11.0)	
Treatment				0.899			0.761
Neoadjuvant_therapy (Cisplatin + Fluorouracil)	91 (15.3)	67 (15.5)	24 (14.7)		27	24	
Surgery	505 (84.7)	366 (84.5)	139 (85.3)		136	139	

*Note:* Data are presented as *n* (%) unless stated. High I‐TILs and P‐TILs were defined as patients with both I‐TILs count and P‐TILs count exceeding the cutoff value. Patients who did not meet the criteria were considered to have low I‐TILs and P‐TILs.

Abbreviations: I‐TILs, intratumor infiltrating lymphocytes; IQR, interquartile range; PSM, propensity score matching; P‐TILs, peritumoral infiltrating lymphocytes.

### 3.2 Determination of the Optimal Combination of TILs

**FIGURE 3:**
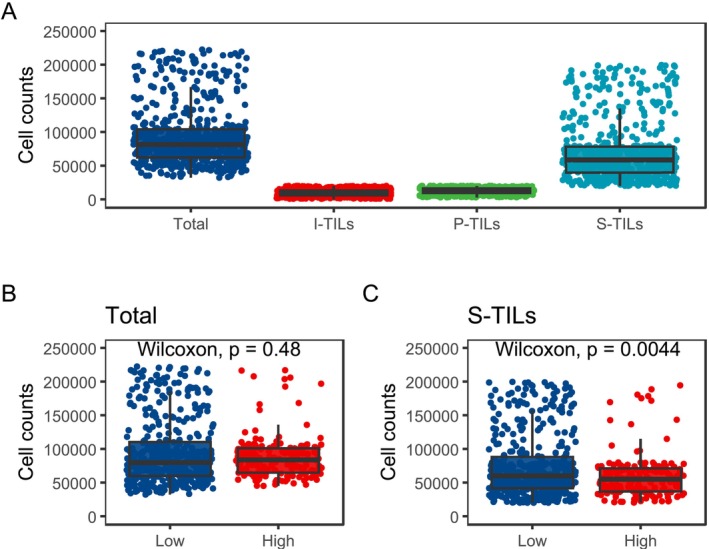
Cell counts distribution of TILs. (A) Distribution of TILs, S‐TILs, I‐TILs, and P‐TILs. (B, C) Distribution of (B) TILs and (C) S‐TILs according to I‐TIL and P‐TIL levels. I‐TILs, intratumor infiltrating lymphocytes; P‐TILs, peritumoral infiltrating lymphocytes; S‐TILs, Stomal tumor infiltrating lymphocytes; TILs, Tumor infiltrating lymphocytes.

Figure 3A illustrates the distribution of TILs. The average counts for total‐TILs, I‐TILs, P‐TILs, and S‐TILs were 93,061.3 ± 45,584.5, 9566.1 ± 4806.3, 12,687.6 ± 4500.9, and 70,807.6 ± 45,095.1, respectively. Optimal cutoff values determined by X‐tiles were 9377, 9483, and 33,580 for I‐TILs, P‐TILs, and S‐TILs, respectively. We defined patients as high I‐TILs and P‐TILs if their counts of I‐TILs and P‐TILs exceeded the cutoff value (Figure 1). Based on this criterion, 27.3% of patients were divided into the high I‐TILs and P‐TILs group, while the rest were in the low I‐TILs and P‐TILs group. No statistically significant difference was observed in total TILs between the two groups (*p* = 0.48, Figure 3B). However, the counts of S‐TILs are significantly lower in the high I‐TILs and P‐TILs group (*p* = 0.0044, Figure 3C). Our analysis revealed no significant difference in the demographic and clinical characteristics of patients, both before and after PSM (Table 1). We also explored other combinations of TILs, but none of them showed a satisfactory capacity to differentiate patients' prognosis (Table S1).

Furthermore, we independently carried out a subgroup analysis with respect to I‐TILs and P‐TILs (Figure S1).

### 3.3 Prognostic Significance of I‐TILs and P‐TILs

**FIGURE 4:**
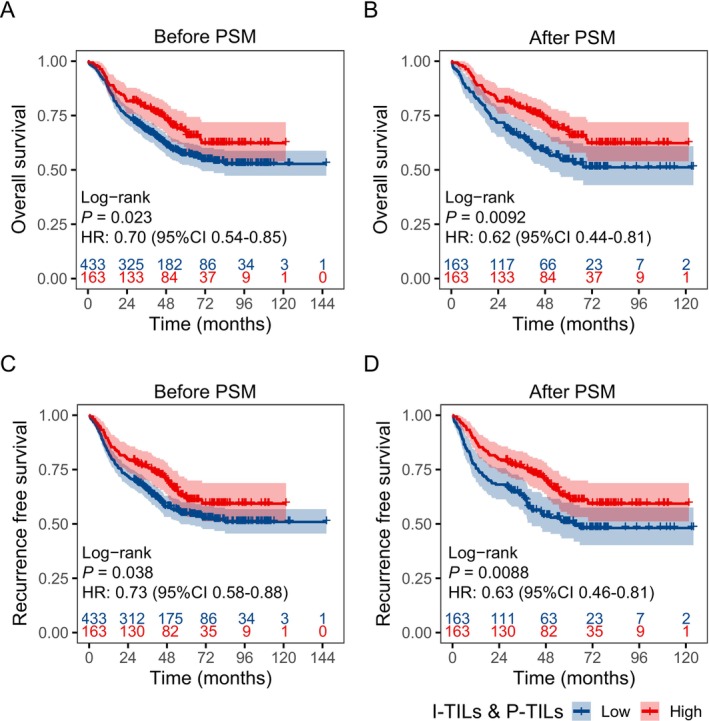
Kaplan–Meier plots for OS and RFS for patients with different I‐TIL and P‐TIL levels. (A) Kaplan–Meier plots for OS before PSM. (B) Kaplan–Meier plots for OS after PSM. (C) Kaplan–Meier plots for RFS before PSM. (D) Kaplan–Meier plots for RFS after PSM. I‐TILs, intratumor infiltrating lymphocytes; OS, overall survival; PSM, propensity score matching; P‐TILs, peritumoral infiltrating lymphocytes; RFS, recurrence‐free survival.

High I‐TILs and P‐TILs were significantly associated with better OS (*p* = 0.023) and RFS (*p* = 0.038) in ESCC patients (Figure 4A,C), which conferred an overall survival hazard ratio (HR) of 0.70 (95% CI: 0.51–0.95, *p* = 0.023) and an RFS HR of 0.73 (95% CI: 0.54–0.98, *p* = 0.039). After PSM, a total of 326 patients were included (Table 1). Twenty‐five percent of them were female and 74.8% of them were male, with a median age of 60 (IQR: 55–65) years old. Demographic and clinical characteristics were well balanced between the groups. Patients with high I‐TILs and P‐TILs had a 5‐year OS of 67.9% (95% CI: 60.5%–76.2%), whereas those with low I‐TILs and P‐TILs had a 5‐year OS of 54.4% (95% CI: 46.8%–63.3%). The group survival difference was statistically significant (*p* = 0.009, Figure 4B). Similar results were observed for 5‐year RFS (high I‐TILs and P‐TILs vs. low I‐TILs and P‐TILs: 62.1% vs. 50.9%, *p* = 0.009, Figure 4D). Multivariable Cox proportional hazards regression further supports this conclusion and recognized I‐TILs and P‐TILs as an independent prognostic factor for OS (HR: 0.63, 95% CI: 0.44–0.91, *p* = 0.014) and RFS (HR: 0.63, 95% CI: 0.45–0.90, *p* = 0.010) after adjusting for age, sex, lymph node invasion, pathologic T stage, histological grade, LVI, resection margin, and treatment (Figure S3). We also conducted a separate analysis of the correlations between I‐TILs or P‐TILs and OS as well as PFS (Figure S2).

### 3.4 Subgroup Analysis

**FIGURE 5:**
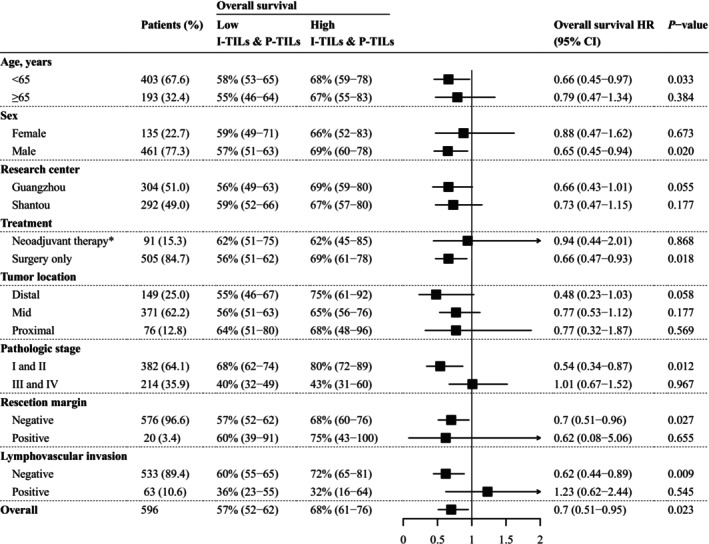
Forest plot for 5‐year overall survival hazard ratios. High I‐TILs and P‐TILs were defined as patients with both intratumor infiltrating lymphocyte count and peritumoral infiltrating lymphocyte count exceeding the cutoff value. Patients who did not meet the criteria were considered to have low I‐TILs and P‐TILs. *Patients in the neoadjuvant therapy subgroup received platinum and fluorouracil‐based neoadjuvant therapy, along with other antitumor agents such as targeted therapy or immunotherapy according to their toleration and gene mutation status. HR, hazard ratios; I‐TILs, intratumor infiltrating lymphocytes; P‐TILs, peritumoral infiltrating lymphocytes.

Subgroup analysis was conducted to validate the general analysis results and identify potential sources of heterogeneity. The direction of the effect size remained consistent in the majority of the subgroups (Figure 5). However, higher levels of I‐TILs and P‐TILs were statistically insignificant for OS in the neoadjuvant therapy (HR: 0.94, 95% CI: 0.44–2.01, *p* = 0.868) and pathologic stage III–IV subgroups (HR: 1.01, 95% CI: 0.67–1.52, *p* = 0.967). Surprisingly, among the 63 patients with LVI, a higher level of I‐TILs and P‐TILs was considered a risk factor (HR: 1.23, 95% CI: 0.62–2.44, *p* = 0.545) for OS.

## 4 Discussion

In this study, we use a DL method to access the counts and distribution of TILs in 597 H&E stained samples from patients with ESCC. Utilizing this data, we categorized patients with both I‐TILs and P‐TILs exceeding the predefined cutoff value as the high I‐TILs and P‐TILs group. Subsequently, we confirmed the predictive significance of I‐TILs and P‐TILs by survival analysis and Cox proportional hazards regression models. These findings suggest that I‐TILs and P‐TILs might serve as a potential favorable prognostic factor in patients with ESCC who received esophagectomy and might provide evidence for determining postoperative treatment. Previous studies have investigated the prognostic significance of TILs in different regions for predicting patient outcomes in ESCC. Sudo, Chiba, Lee, Zhou et al. reported that a high level of peritumoral TILs is a strong predictor of a better prognosis in ESCC patients [31, 32, 33, 34]. Liu et al. [35] discovered that in patients with colorectal cancer (CRC), a higher density of both intratumoral and peritumoral TILs is associated with a more favorable progression‐free survival (PFS), whereas stromal TILs demonstrate no significant association with PFS. Khoury et al. [36] propose that in triple‐negative breast cancer (TNBC), TILs across all regions are strongly correlated with pathological complete response (pCR). Hao et al., in a meta‐analysis, proposed that high levels of general TILs, rather than I‐TILs or P‐TILs alone, are associated with better OS and DFS in ESCC patients [12]. However, there are also studies holding opposite views. In the research on patients with locally advanced esophageal cancer receiving radiotherapy and immunotherapy, Zhang et al. concluded that general TILs were not associated with patients' OS and PFS [13]. The association between the count of I‐TILs and P‐TILs and prognosis can be explained from several biological perspectives. First, TILs play a pivotal role in immune surveillance. Consisting of diverse immune cells such as CD8^+^ cytotoxic T lymphocytes (CTLs) and CD4^+^ helper T cells, they are capable of identifying and eliminating tumor cells by zeroing in on tumor‐associated antigens. A higher count of TILs generally indicates more effective suppression of tumor growth. Second, in the tumor microenvironment, TILs secrete cytokines (IFN‐γ, TNF‐α) to modulate immune responses and have an impact on angiogenesis. This helps to reshape the environment in a way that is less conducive to tumor growth. In addition, they work in concert with antigen‐presenting cells and maintain a delicate balance with regulatory T lymphocytes, all of which contribute to strengthening the antitumor effect. In some studies, it has been demonstrated that the general tumor‐infiltrating lymphocytes (TILs) exhibit no correlation with the prognosis of patients. This implies that the association between TILs and prognosis might be contingent upon the differential regional distributions of TILs. Our research primarily substantiates that the prognosis of patients is associated with peritumoral TILs and intratumoral TILs, yet is independent of stromal TILs. Notwithstanding, stromal TILs constitute a substantial proportion within immune cells. When deliberating upon the general TILs, the preponderant weight of stromal TILs may engender the outcome wherein the general TILs bear no relation to prognosis. Overall, the prognostic value of TILs in different regions for ESCC patients is still controversial [37]. Employing a deep learning approach, we have identified that both intratumoral TILs and peritumoral TILs possess prognostic implications. Our research further delved into the prognostic significance of diverse combinations of TILs and ultimately ascertained that the I‐TILs and P‐TILs based on deep learning represents a more reliable and stable prognostic indicator. In our study, lower S‐TIL levels were found in high I‐TIL and P‐TIL patients, which may refer to a recruit of TILs to the center of the tumor region. The tumor‐peripheral stroma encompasses a multiplicity of immune and nonimmune cellular constituents. Immune cells engage in reciprocal interactions through the secretion of cytokines. Notably, chemokines like CXCL13 within the stroma are capable of instigating the migration of lymphocytes towards the infiltrative margin [17]. Based on the existing evidence, we tend to believe that high levels of I‐TILs and P‐TILs, rather than S‐TILs, are associated with the favorable prognosis of ESCC patients. Additionally, a significantly high level of TILs is necessary to improve patient prognosis. Therefore, the cut‐off values of both I‐TILs and P‐TILs need to be used concurrently to distinguish patients with better prognosis in this study. Future research should validate these findings in a larger sample size and further explore the underlying mechanisms.

Several interesting findings emerged from the stratified analysis. In patients who underwent surgery only, intraepithelial TILs (I‐TILs) and peritumoral TILs (P‐TILs) showed significant predictive value. However, this association was not observed in patients who received neoadjuvant therapy. This disparity could be attributed to the comparatively low cut‐off value used in this study, which was derived from the entire sample population [38, 39]. This cut‐off value may be too low for patients who develop an immune response after neoadjuvant treatment, thereby affecting its effectiveness in this patient subset. Therefore, independent prognostic factors and separate cut‐off values may be necessary for this specific patient subset. Similarly, I‐TILs & P‐TILs hold prognostic significance for patients in stages I–II but exhibit poor performance for patients in stages III‐IV. There is considerable overlap between patients in stages III‐IV and those receiving neoadjuvant treatment (51.6%). Previous research has shown that patients at more advanced stages tend to have higher levels of TILs compared to those at early stages [12]. As mentioned above, this could potentially affect the diagnostic efficacy of the cut‐off value. Therefore, conducting focused research targeting different stages of the disease is a crucial direction for future investigations. Finally, the association between high levels of I‐TILs and P‐TILs and poor prognosis in the LVI‐positive subgroup is a finding with marked heterogeneity, although it is not statistically significant. Limited studies have directly focused on the relationship between TILs and LVI. It has been suggested that positive LVI is associated with a high level of systemic immune‐inflammation index, a new inflammatory index that reflects the inflammatory status [40]. Previous research has also found a significant correlation between systemic immune‐inflammation index and TILs in tumor tissues [41, 42, 43]. Even so, robust conclusions are challenging due to the available evidence. We can only speculate that a higher level of immune infiltrating may be positively associated with the degree of LVI in the LVI‐positive subgroup, resulting in an association between high I‐TILs and P‐TILs and poor prognosis. Further studies are needed to confirm our speculation.

DL has played an indispensable role in this study, allowing us to determine the boundaries of tumors and count individual TILs accurately and quickly. As far as we know, there is currently limited research applying DL in ESCC [3, 44]. Considering the efficiency and consistent performance of AI technology in image recognition, continuous attention and research in this field are still necessary [45, 46].

Some limitations of this study should be acknowledged. First, the prognostic significance of lymphocyte subgroups was not investigated, as only HE‐stained samples were included. Additionally, the utilization of spatial information in this study is limited. Although we have identified the distribution of TILs in different tissue regions, further investigation is warranted to examine their distribution within these regions. For instance, it would be valuable to determine whether TILs exhibit a higher clustering tendency in the center or at the periphery of the tumor. Furthermore, in light of the inadequate support from computing power and algorithms, this research refrained from normalizing the measured values with respect to area and ultimately presented the results in the form of cell density. Last, the conclusion from subgroup analyses should be interpreted with caution because of the limitations of the data.

## 5 Conclusion

In the present study, we revealed that I‐TILs and P‐TILs serve as independent prognostic factors for ESCC patients using DL approaches. Further study should concentrate on the prognostic value of lymphocyte subgroups and make better use of the spatial information to improve the predictive efficacy of TILs.

## Author Contributions

**Peishen Li:** formal analysis (equal), methodology (equal), visualization (equal), writing – original draft (equal), writing – review and editing (equal). **Shujie Huang:** conceptualization (equal), data curation (equal), formal analysis (equal), methodology (equal), visualization (equal), writing – original draft (equal), writing – review and editing (equal). **Haijie Xu:** formal analysis (equal), methodology (equal), visualization (equal), writing – original draft (equal), writing – review and editing (equal). **Zijie Li:** data curation (equal), investigation (equal). **Sichao Wang:** data curation (equal), investigation (equal). **Zhen Gao:** data curation (equal), software (equal). **Yuejiao Dong:** data curation (equal). **Zhuofeng Chen:** data curation (equal). **Guibin Qiao:** writing – review and editing (equal). **Hansheng Wu:** project administration (equal), supervision (equal), writing – review and editing (equal). **Liangli Hong:** conceptualization (equal), project administration (equal), supervision (equal), writing – review and editing (equal).

## Ethics Statement

This study was authorized by the ethics committee of the GDPH (No. GDREC2020142H) and the SUMCFH (No. 2020‐094). Written informed consent was obtained from all the patients. This study was conducted under the Declaration of Helsinki (as revised in 2013).

## Conflicts of Interest

The authors declare no conflicts of interest.

## Supporting information


**Figure S1.** Cell counts distribution of TILs with different I‐TIL and P‐TIL levels. (A) Distribution of TILs according to I‐TIL and P‐TIL levels. (B) Distribution of S‐TILs according to I‐TIL and P‐TIL levels. I‐TILs, intratumor infiltrating lymphocytes; P‐TILs, peritumoral infiltrating lymphocytes; S‐TILs, stomal tumor infiltrating lymphocytes; TILs, tumor infiltrating lymphocytes.

**Figure S2.** Kaplan–Meier plots for OS and RFS for patients with different I‐TIL and P‐TIL levels. (A) Kaplan–Meier plots for OS with different I‐TIL and P‐TIL levels. (B) Kaplan–Meier plots for RFS with different I‐TIL and P‐TIL levels. I‐TILs, intratumor infiltrating lymphocytes; OS, overall survival; PSM, propensity score matching; P‐TILs, peritumoral infiltrating lymphocytes; RFS, recurrence‐free survival.

**Figure S3.** Forest plot for multivariate COX proportional hazards regression analysis. Figure S3 presents the hazard ratios (HR) and corresponding *p* values from the multivariate COX regression model incorporating clinical variables (TumorL_AroundL, age, sex, lymph node invasion (pN), pathologic T stage (pT), histological grade (G), LVI, resection margin (R0), and treatment) to assess their impact on survival. TumorL_AroundL: intratumor infiltrating lymphocytes and peritumoral infiltrating lymphocytes.

**Table S1.** Selection of prognostic factor for esophageal squamous cell carcinoma.

## Data Availability

The data supporting the findings of this study are available from the corresponding author upon request via email (Email: wu-han-sheng@163.com).

## References

[1] R. L. Siegel , K. D. Miller , N. S. Wagle , and A. Jemal , “Cancer Statistics, 2023,” CA: A Cancer Journal for Clinicians 73, no. 1 (2023): 17–48, 10.3322/caac.21763. 36633525

[2] NCCN Guidelines for Patients: Esophageal Cancer , “Esophageal Cancer” (2022), https://www.nccn.org/patients/guidelines/content/PDF/esophageal‐patient.pdf.

[3] K. Kouzu , I. P. Nearchou , Y. Kajiwara , et al., “Deep‐Learning‐Based Classification of Desmoplastic Reaction on H&E Predicts Poor Prognosis in Oesophageal Squamous Cell Carcinoma,” Histopathology 81, no. 2 (2022): 255–263, 10.1111/his.14708. 35758184

[4] S. Sasagawa , H. Kato , K. Nagaoka , et al., “Immuno‐Genomic Profiling of Biopsy Specimens Predicts Neoadjuvant Chemotherapy Response in Esophageal Squamous Cell Carcinoma,” Cell Reports Medicine 3, no. 8 (2022): 100705, 10.1016/j.xcrm.2022.100705. 35944530 PMC9418738

[5] J. Li , Y. Tang , L. Huang , et al., “A High Number of Stromal Tumor‐Infiltrating Lymphocytes Is a Favorable Independent Prognostic Factor in M0 (Stages I–III) Esophageal Squamous Cell Carcinoma,” Diseases of the Esophagus 30, no. 1 (2017): 1–7, 10.1111/dote.12518. 27868286

[6] H. Y. Li , M. McSharry , B. Bullock , et al., “The Tumor Microenvironment Regulates Sensitivity of Murine Lung Tumors to PD‐1/PD‐L1 Antibody Blockade,” Cancer Immunology Research 5, no. 9 (2017): 767–777, 10.1158/2326-6066.CIR-16-0365. 28819064 PMC5787226

[7] Y. Zheng , Y. Li , B. Tang , et al., “IL‐6‐Induced CD39 Expression on Tumor‐Infiltrating NK Cells Predicts Poor Prognosis in Esophageal Squamous Cell Carcinoma,” Cancer Immunology, Immunotherapy 69, no. 11 (2020): 2371–2380, 10.1007/s00262-020-02629-1. 32524362 PMC11027717

[8] T. Mori , K. Kumagai , K. Nasu , et al., “Clonal Expansion of Tumor‐Infiltrating T Cells and Analysis of the Tumor Microenvironment Within Esophageal Squamous Cell Carcinoma Relapsed After Definitive Chemoradiation Therapy,” International Journal of Molecular Sciences 22, no. 3 (2021): 1098, 10.3390/ijms22031098. 33499345 PMC7865796

[9] K. Chen , Z. Zhu , N. Zhang , et al., “Tumor‐Infiltrating CD4^+^ Lymphocytes Predict a Favorable Survival in Patients With Operable Esophageal Squamous Cell Carcinoma,” Medical Science Monitor 23 (2017): 4619–4632, 10.12659/msm.904154. 28949934 PMC5687116

[10] D. Qian , Y. Wang , G. Zhao , et al., “Tumor Remission and Tumor‐Infiltrating Lymphocytes During Chemoradiation Therapy: Predictive and Prognostic Markers in Locally Advanced Esophageal Squamous Cell Carcinoma,” International Journal of Radiation Oncology, Biology, Physics 105, no. 2 (2019): 319–328, 10.1016/j.ijrobp.2019.06.079. 31228553

[11] M. Lopez de Rodas , V. Nagineni , A. Ravi , et al., “Role of Tumor Infiltrating Lymphocytes and Spatial Immune Heterogeneity in Sensitivity to PD‐1 Axis Blockers in Non‐Small Cell Lung Cancer,” Journal for Immunotherapy of Cancer 10, no. 6 (2022): e004440, 10.1136/jitc-2021-004440. 35649657 PMC9161072

[12] J. Hao , M. Li , T. Zhang , et al., “Prognostic Value of Tumor‐Infiltrating Lymphocytes Differs Depending on Lymphocyte Subsets in Esophageal Squamous Cell Carcinoma: An Updated Meta‐Analysis,” Frontiers in Oncology 10 (2020): 614, 10.3389/fonc.2020.00614. 32411602 PMC7198736

[13] W. Zhang , C. Yan , X. Gao , et al., “Safety and Feasibility of Radiotherapy Plus Camrelizumab for Locally Advanced Esophageal Squamous Cell Carcinoma,” Oncologist 26, no. 7 (2021): e1110–e1124, 10.1002/onco.13797. 33893689 PMC8265339

[14] Z. Li , L. Liu , B. Wang , J. Ying , J. He , and L. Xue , “Tumor Budding and Tumor‐Infiltrating Lymphocytes Can Predict Prognosis in pT1b Esophageal Squamous Cell Carcinoma,” Thoracic Cancer 14 (2023): 2608–2617, 10.1111/1759-7714.15043. 37466146 PMC10481137

[15] Z. Ren , A. Zhang , Z. Sun , et al., “Selective Delivery of Low‐Affinity IL‐2 to PD‐1^+^ T Cells Rejuvenates Antitumor Immunity With Reduced Toxicity,” Journal of Clinical Investigation 132, no. 3 (2022): e153604, 10.1172/JCI153604. 35104810 PMC8803347

[16] Y. Chu , J. Liao , J. Li , et al., “CD103^+^ Tumor‐Infiltrating Lymphocytes Predict Favorable Prognosis in Patients With Esophageal Squamous Cell Carcinoma,” Journal of Cancer 10, no. 21 (2019): 5234–5243, 10.7150/jca.30354. 31602274 PMC6775603

[17] S. T. Paijens , A. Vledder , M. de Bruyn , and H. W. Nijman , “Tumor‐Infiltrating Lymphocytes in the Immunotherapy Era,” Cellular & Molecular Immunology 18, no. 4 (2021): 842–859, 10.1038/s41423-020-00565-9. 33139907 PMC8115290

[18] H. Xu , Y. J. Cha , J. R. Clemenceau , et al., “Spatial Analysis of Tumor‐Infiltrating Lymphocytes in Histological Sections Using Deep Learning Techniques Predicts Survival in Colorectal Carcinoma,” Journal of Pathology. Clinical Research 8, no. 4 (2022): 327–339, 10.1002/cjp2.273. 35484698 PMC9161341

[19] J. Saltz , R. Gupta , L. Hou , et al., “Spatial Organization and Molecular Correlation of Tumor‐Infiltrating Lymphocytes Using Deep Learning on Pathology Images,” Cell Reports 23, no. 1 (2018): 181–193.e7, 10.1016/j.celrep.2018.03.086. 29617659 PMC5943714

[20] Z. Zhou , M. M. R. Siddiquee , N. Tajbakhsh , and J. Liang , “UNet++: Redesigning Skip Connections to Exploit Multiscale Features in Image Segmentation,” IEEE Transactions on Medical Imaging 39, no. 6 (2020): 1856–1867, 10.1109/TMI.2019.2959609. 31841402 PMC7357299

[21] Z. Zhou , M. M. R. Siddiquee , N. Tajbakhsh , and J. Liang , “UNet++: A Nested U‐Net Architecture for Medical Image Segmentation,” Deep Learning in Medical Image Analysis and Multimodal Learning for Clinical Decision Support: 4th International Workshop, DLMIA 2018, and 8th International Workshop, ML‐CDS 2018, Held in Conjunction With MICCAI 2018, Granada, Spain 11045 (2018): 3–11, 10.1007/978-3-030-00889-5_1. PMC7329239 32613207

[22] G. Li , V. Sanchez , G. Patel , S. Quenby , and N. Rajpoot , “Localisation of Luminal Epithelium Edge in Digital Histopathology Images of IHC Stained Slides of Endometrial Biopsies,” Computerized Medical Imaging and Graphics 42 (2015): 56–64, 10.1016/j.compmedimag.2014.11.007. 25529641

[23] A. Haghofer , A. Fuchs‐Baumgartinger , K. Lipnik , et al., “Histological Classification of Canine and Feline Lymphoma Using a Modular Approach Based on Deep Learning and Advanced Image Processing,” Scientific Reports 13, no. 1 (2023): 19436, 10.1038/s41598-023-46607-w. 37945699 PMC10636139

[24] A. D. Nelson and S. Krishna , “An Effective Approach for the Nuclei Segmentation From Breast Histopathological Images Using Star‐Convex Polygon,” Procedia Computer Science 218 (2023): 1778–1790, 10.1016/j.procs.2023.01.156.

[25] Y. Zhao , C. Fu , W. Zhang , C. Ye , Z. Wang , and H. F. Ma , “Automatic Segmentation of Cervical Cells Based on Star‐Convex Polygons in Pap Smear Images,” Bioengineering (Basel) 10, no. 1 (2022): 47, https://pubmed.ncbi.nlm.nih.gov/36671619/. 36671619 10.3390/bioengineering10010047 PMC9854569

[26] K. He , X. Chen , S. Xie , Y. Li , P. Dollár , and R. Girshick , “Masked Autoencoders Are Scalable Vision Learners” (2021), 10.48550/arXiv.2111.06377.

[27] K. He , X. Zhang , S. Ren , and J. Sun , “Deep Residual Learning for Image Recognition” (2015), 10.48550/arXiv.1512.03385.

[28] A. Saini , K. Guleria , and S. Sharma , “Optimizing White Blood Cell Classification With ResNet50 Neural Network,” in 2024 IEEE International Conference on Communication, Computing and Signal Processing (IICCCS) (IEEE, 2024), 1–6, 10.1109/IICCCS61609.2024.10763643.

[29] S. Sornapudi , G. T. Brown , Z. Xue , R. Long , L. Allen , and S. Antani , “Comparing Deep Learning Models for Multi‐Cell Classification in Liquid‐Based Cervical Cytology Image,” AMIA Annual Symposium proceedings. AMIA Symposium 2019 (2019): 820–827. 32308878 PMC7153123

[30] R Core Team R , “R: A Language and Environment for Statistical Computing” (2013), https://apps.dtic.mil/sti/citations/AD1039033.

[31] T. Sudo , R. Nishida , A. Kawahara , et al., “Clinical Impact of Tumor‐Infiltrating Lymphocytes in Esophageal Squamous Cell Carcinoma,” Annals of Surgical Oncology 24, no. 12 (2017): 3763–3770, 10.1245/s10434-017-5796-4. 28160141

[32] T. Chiba , H. Ohtani , T. Mizoi , et al., “Intraepithelial CD8^+^ T‐Cell‐Count Becomes a Prognostic Factor After a Longer Follow‐Up Period in Human Colorectal Carcinoma: Possible Association With Suppression of Micrometastasis,” British Journal of Cancer 91, no. 9 (2004): 1711–1717, 10.1038/sj.bjc.6602201. 15494715 PMC2410024

[33] H. J. Lee , I. A. Park , I. H. Song , et al., “Tertiary Lymphoid Structures: Prognostic Significance and Relationship With Tumour‐Infiltrating Lymphocytes in Triple‐Negative Breast Cancer,” Journal of Clinical Pathology 69, no. 5 (2016): 422–430, https://pubmed.ncbi.nlm.nih.gov/26475777/. 26475777 10.1136/jclinpath-2015-203089

[34] C. Zhou , Y. Wu , L. Jiang , et al., “Density and Location of CD3^+^ and CD8^+^ Tumor‐Infiltrating Lymphocytes Correlate With Prognosis of Oral Squamous Cell Carcinoma,” Journal of Oral Pathology & Medicine 47, no. 4 (2018): 359–367, https://pubmed.ncbi.nlm.nih.gov/29469989/. 29469989 10.1111/jop.12698

[35] L. Liu , M. Long , S. Su , L. Wang , and J. Liu , “Clinical Impact of Heterogeneously Distributed Tumor‐Infiltrating Lymphocytes on the Prognosis of Colorectal Cancer,” PeerJ 12 (2024): e16747, 10.7717/peerj.16747. 38223758 PMC10785792

[36] T. Khoury , V. Nagrale , M. Opyrchal , X. Peng , D. Wang , and S. Yao , “Prognostic Significance of Stromal Versus Intratumoral Infiltrating Lymphocytes in Different Subtypes of Breast Cancer Treated With Cytotoxic Neoadjuvant Chemotherapy,” Applied Immunohistochemistry & Molecular Morphology 26, no. 8 (2018): 523–532, 10.1097/PAI.0000000000000466. 28187033 PMC5550367

[37] Y. Baba , D. Nomoto , K. Okadome , et al., “Tumor Immune Microenvironment and Immune Checkpoint Inhibitors in Esophageal Squamous Cell Carcinoma,” Cancer Science 111, no. 9 (2020): 3132–3141, 10.1111/cas.14541. 32579769 PMC7469863

[38] S. Deguchi , H. Tanaka , S. Suzuki , et al., “Clinical Relevance of Tertiary Lymphoid Structures in Esophageal Squamous Cell Carcinoma,” BMC Cancer 22 (2022): 699, 10.1186/s12885-022-09777-w. 35751038 PMC9233387

[39] E. Fukuoka , K. Yamashita , T. Tanaka , et al., “Neoadjuvant Chemotherapy Increases PD‐L1 Expression and CD8^+^ Tumor‐Infiltrating Lymphocytes in Esophageal Squamous Cell Carcinoma,” Anticancer Research 39, no. 8 (2019): 4539–4548, 10.21873/anticanres.13631. 31366557

[40] Y. Ji and H. Wang , “Prognostic Prediction of Systemic Immune‐Inflammation Index for Patients With Gynecological and Breast Cancers: A Meta‐Analysis,” World Journal of Surgical Oncology 18, no. 1 (2020): 197, 10.1186/s12957-020-01974-w. 32767977 PMC7414550

[41] K. M , M. N , E. P , et al., “Lymphovascular Invasion Is Associated With Mutational Burden and PD‐L1 in Resected Lung Cancer,” Annals of Thoracic Surgery 109, no. 2 (2020): 358–366, 10.1016/j.athoracsur.2019.08.029. 31550464 PMC7366360

[42] Q. K. Xie , P. Chen , W. M. Hu , et al., “The Systemic Immune‐Inflammation Index Is an Independent Predictor of Survival for Metastatic Colorectal Cancer and Its Association With the Lymphocytic Response to the Tumor,” Journal of Translational Medicine 16, no. 1 (2018): 273, 10.1186/s12967-018-1638-9. 30286769 PMC6172841

[43] E. Atasever Akkas and B. Yucel , “Prognostic Value of Systemic Immune Inflammation Index in Patients With Laryngeal Cancer,” European Archives of Oto‐Rhino‐Laryngology 278, no. 6 (2021): 1945–1955, 10.1007/s00405-021-06798-2. 33837464

[44] C. Suo , H. Chen , F. Binczyk , et al., “Tumor Infiltrating Lymphocyte Signature Is Associated With Single Nucleotide Polymorphisms and Predicts Survival in Esophageal Squamous Cell Carcinoma Patients,” Aging 13, no. 7 (2021): 10369–10386, 10.18632/aging.202798. 33819921 PMC8064198

[45] Y. She , B. He , F. Wang , et al., “Deep Learning for Predicting Major Pathological Response to Neoadjuvant Chemoimmunotherapy in Non‐Small Cell Lung Cancer: A Multicentre Study,” eBioMedicine 86 (2022): 104364, 10.1016/j.ebiom.2022.104364. 36395737 PMC9672965

[46] N. Coudray , P. S. Ocampo , T. Sakellaropoulos , et al., “Classification and Mutation Prediction From Non‐Small Cell Lung Cancer Histopathology Images Using Deep Learning,” Nature Medicine 24, no. 10 (2018): 1559–1567, 10.1038/s41591-018-0177-5. PMC9847512 30224757

