# Supplementary figures and images for: Prognostic Value of Deep Learning‐Extracted Tumor‐Infiltrating Lymphocytes in Esophageal Cancer: A Multicenter Retrospective Cohort Study
Source: Cancer Med. 2025 Jul 17;14(14):e71054. doi: 10.1002/cam4.71054 (PMC12268311; doi:10.1002/cam4.71054)

A

Total

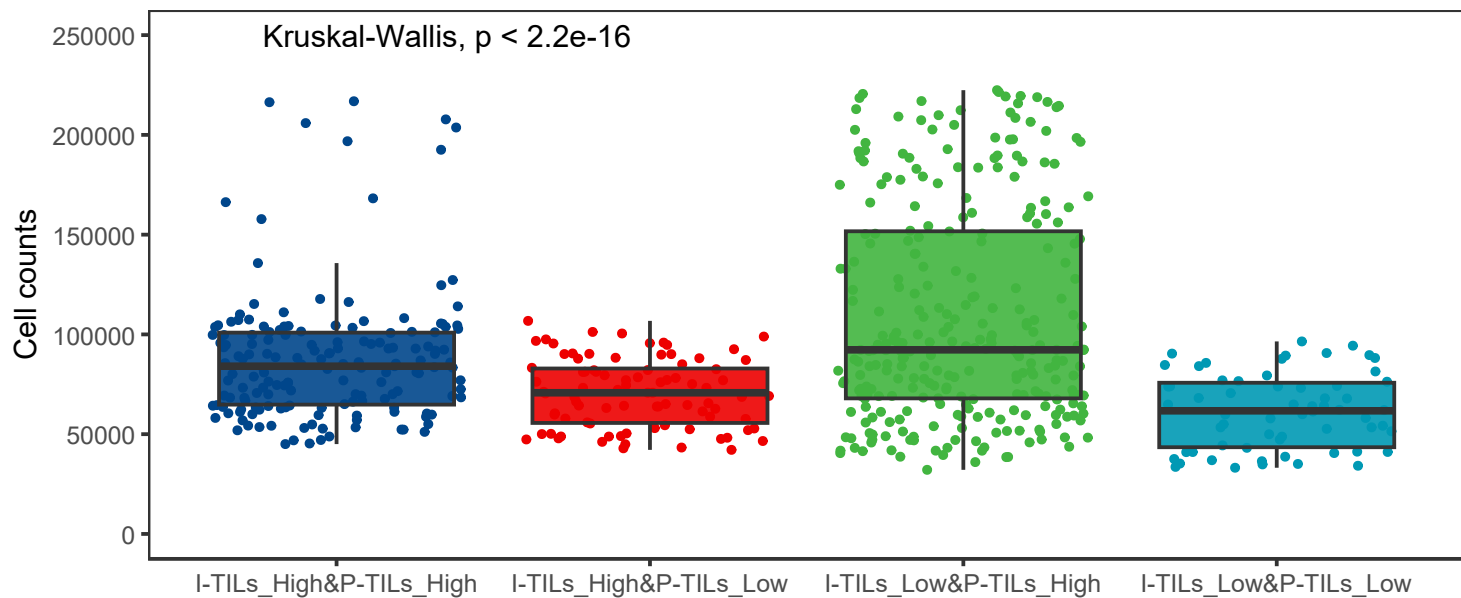

B

S-TILs

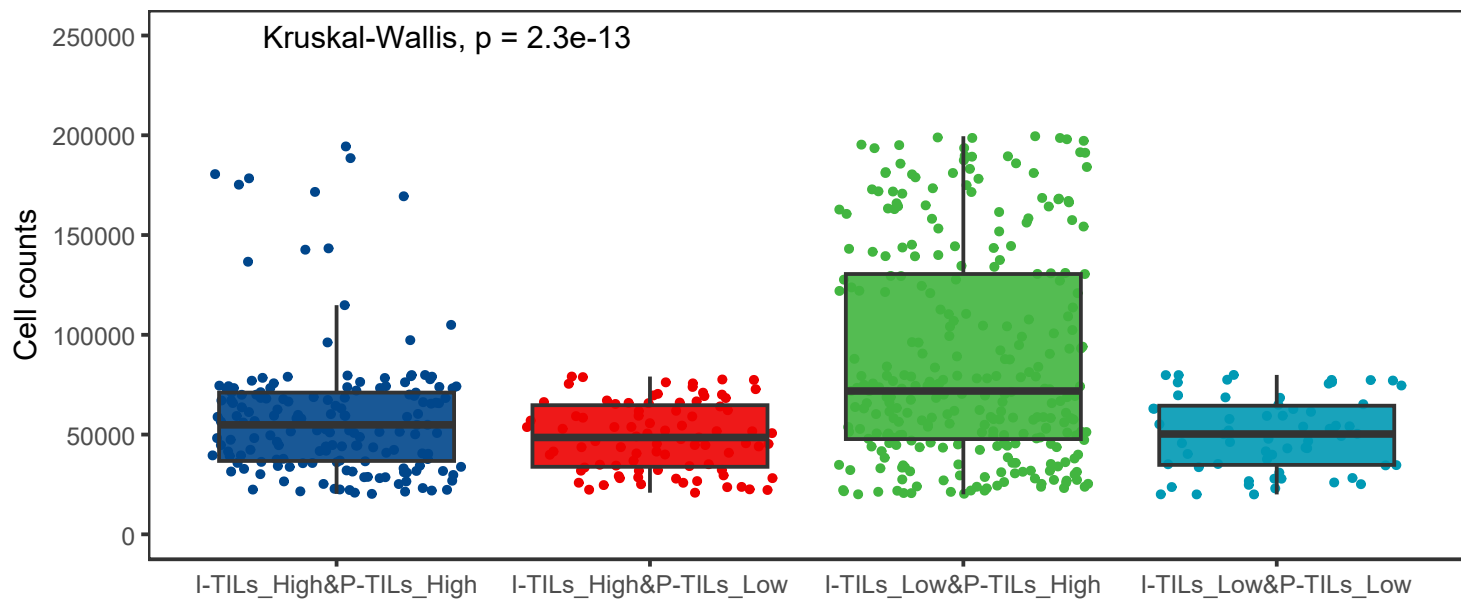

Supplement: Supplementary file 1 — Figure S1. Cell counts distribution of TILs with different I‐TIL and P‐TIL levels. (A) Distribution of TILs according to I‐TIL and P‐TIL levels. (B) Distribution of S‐TILs according to I‐TIL and P‐TIL levels. I‐TILs, intratumor infiltrating lymphocytes; P‐TILs, peritumoral infiltrating lymphocytes; S‐TILs, stomal tumor infiltrating lymphocytes; TILs, tumor infiltrating lymphocytes. [file CAM4-14-e71054-s003.pdf]

A

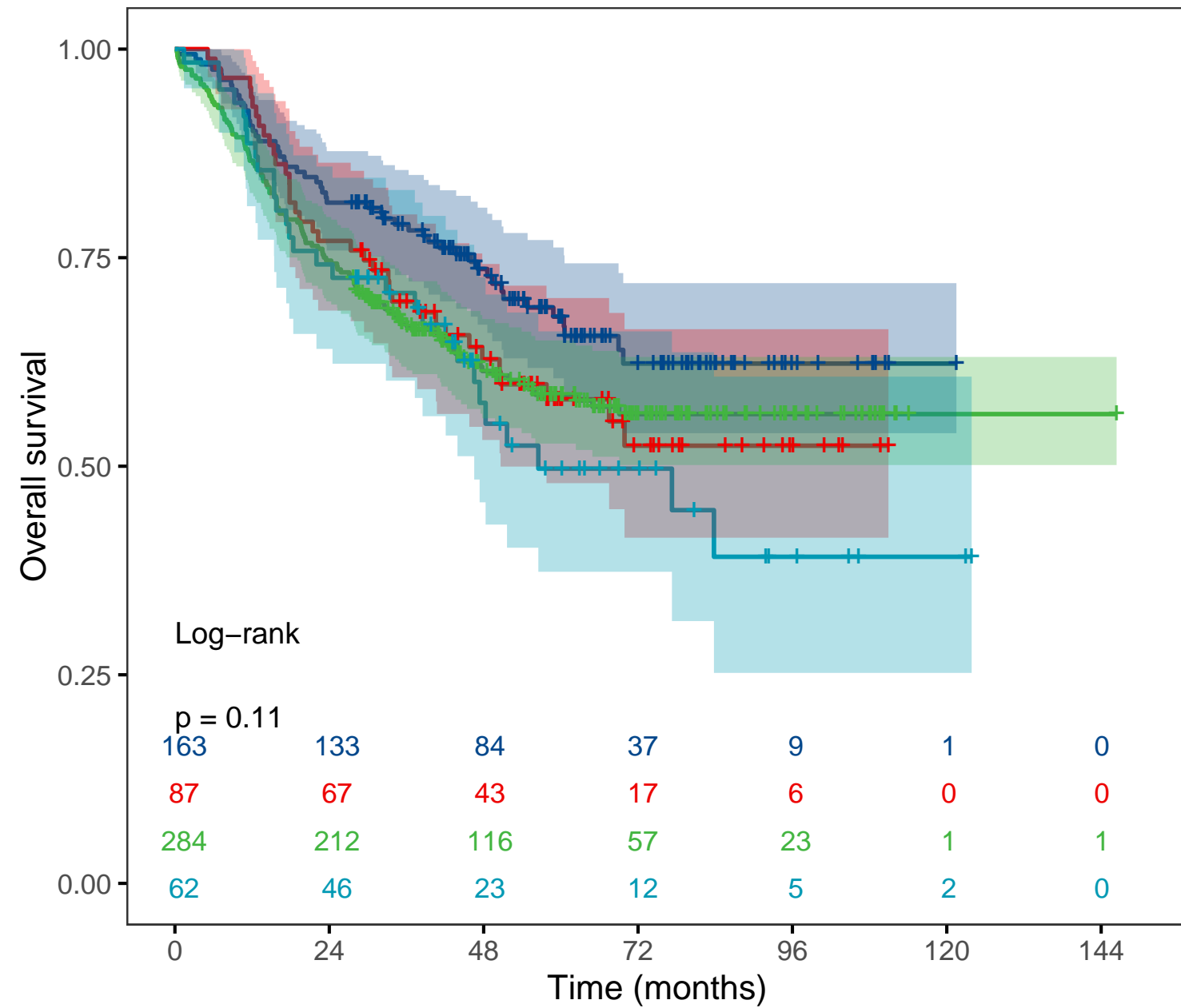

B

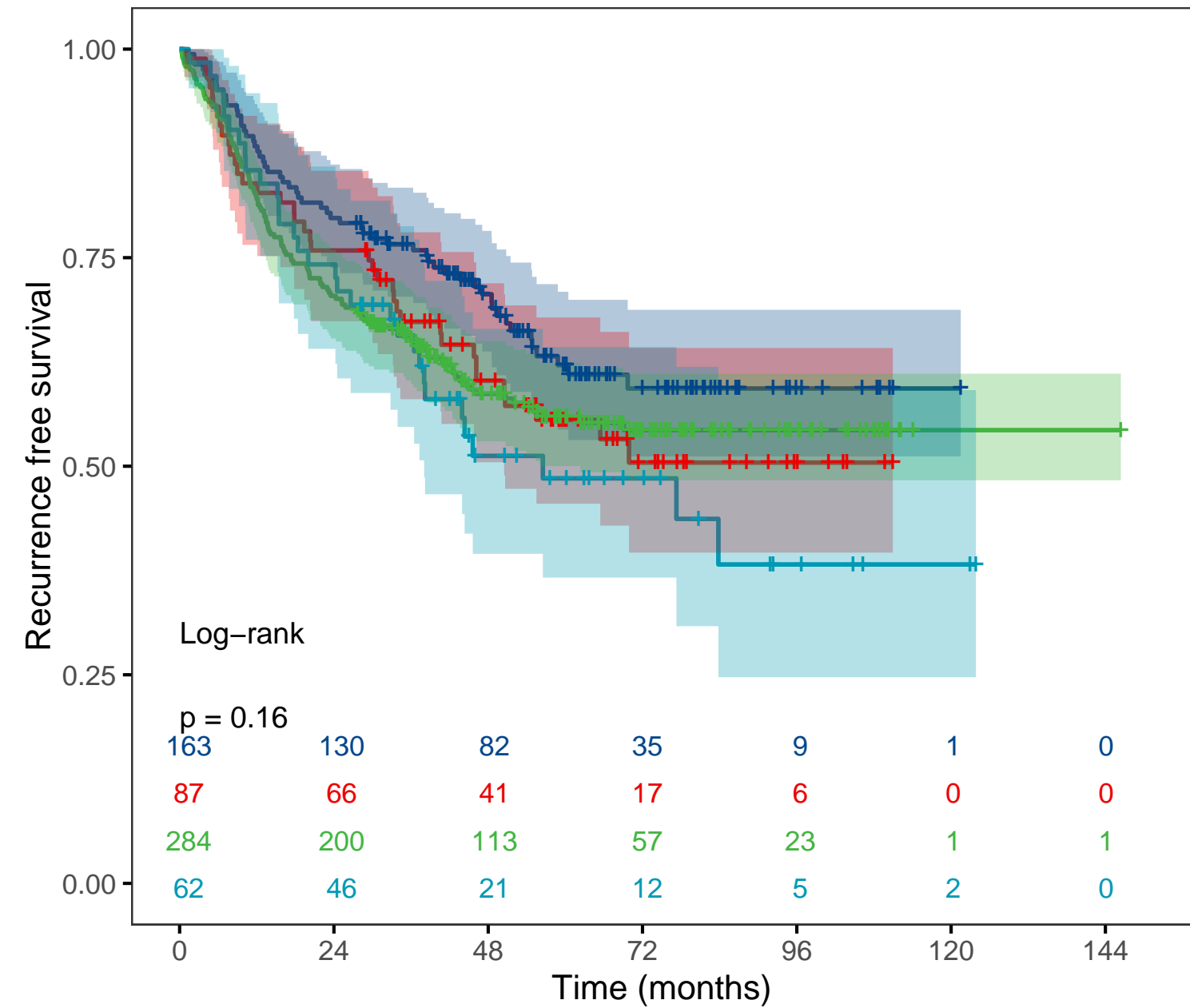

I-TILs & P-TILs High & High Low & Low High & Low Low & High

I-TILs & P-TILs High & High Low & Low High & Low Low & High

Supplement: Supplementary file 2 — Figure S2. Kaplan–Meier plots for OS and RFS for patients with different I‐TIL and P‐TIL levels. (A) Kaplan–Meier plots for OS with different I‐TIL and P‐TIL levels. (B) Kaplan–Meier plots for RFS with different I‐TIL and P‐TIL levels. I‐TILs, intratumor infiltrating lymphocytes; OS, overall survival; PSM, propensity score matching; P‐TILs, peritumoral infiltrating lymphocytes; RFS, recurrence‐free survival. [file CAM4-14-e71054-s004.pdf]

Hazard ratio

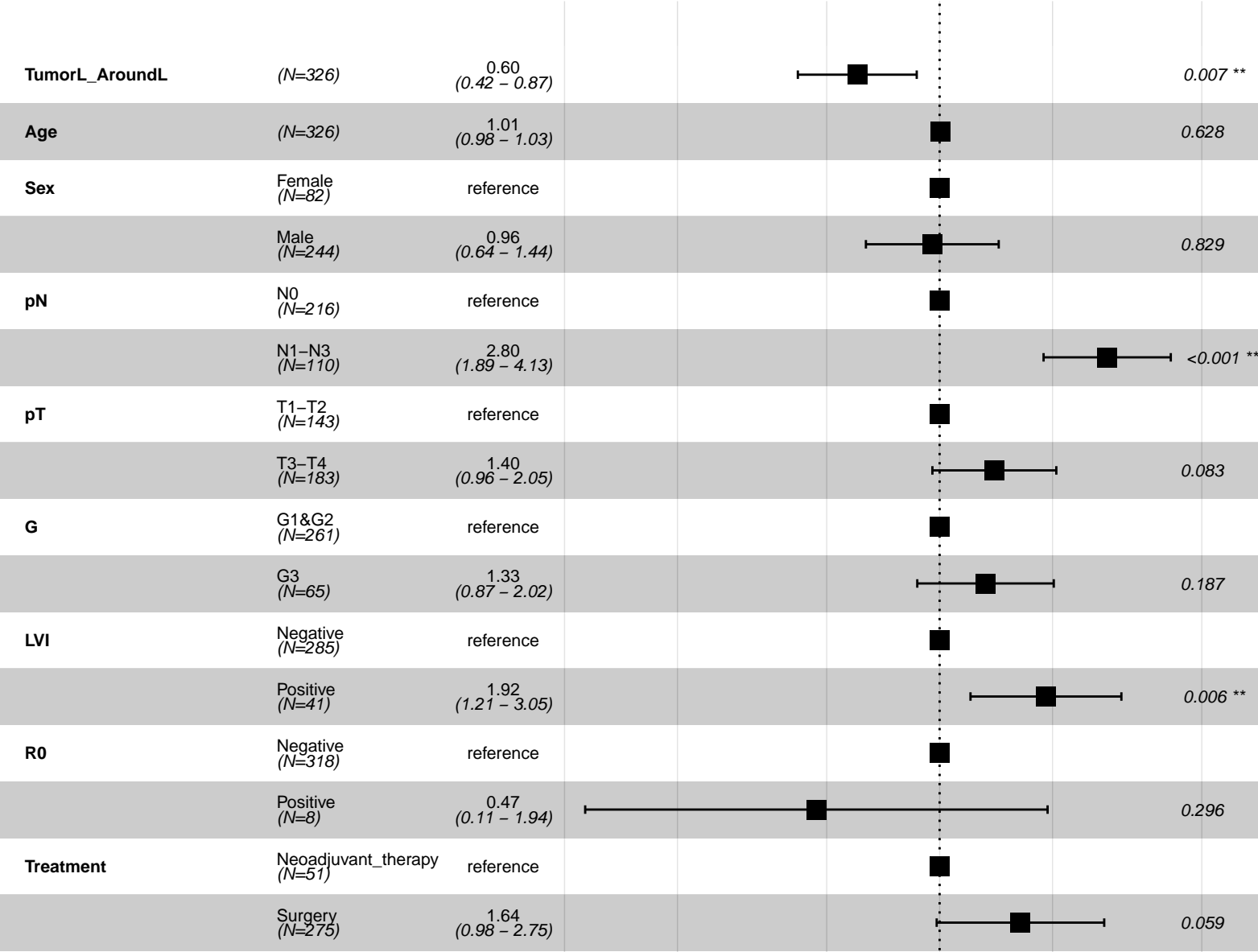

# Events: 123; Global p-value (Log-Rank): 4.5572e-10  
AIC: 1293.29; Concordance Index: 0.72

Supplement: Supplementary file 3 — Figure S3. Forest plot for multivariate COX proportional hazards regression analysis. Figure S3 presents the hazard ratios (HR) and corresponding p values from the multivariate COX regression model incorporating clinical variables (TumorL_AroundL, age, sex, lymph node invasion (pN), pathologic T stage (pT), histological grade (G), LVI, resection margin (R0), and treatment) to assess their impact on survival. TumorL_AroundL: intratumor infiltrating lymphocytes and peritumoral infiltrating lymphocytes. [file CAM4-14-e71054-s002.pdf]
