# Supplementary material for: Prognostic Value of Deep Learning‐Extracted Tumor‐Infiltrating Lymphocytes in Esophageal Cancer: A Multicenter Retrospective Cohort Study
Source: Cancer Med. 2025 Jul 17;14(14):e71054. doi: 10.1002/cam4.71054 (PMC12268311; doi:10.1002/cam4.71054)
Supplement: Supplementary file 4 — Table S1. Selection of prognostic factor for esophageal squamous cell carcinoma. [file CAM4-14-e71054-s001.docx]

**Supplemental Table 1. Selection of prognostic factor for esophageal squamous cell carcinoma**

| **Indicator** | ***P*-value** |
| --- | --- |
| I-TILs | 0.08 |
| P-TILs | 0.19 |
| S-TILs | 0.19 |
| I-TILs/P-TILs | 0.24 |
| I-TILs/S-TILs | 0.25 |
| S-TILs/P-TILs | 0.18 |
| I-TILs & S-TILs | 0.31 |
| S-TILs & P-TILs | 0.45 |

**Note:** I-TILs: intra-tumor infiltrating lymphocytes, P-TILs: peritumoral infiltrating lymphocytes, S-TILs: stomal tumor infiltrating lymphocytes. I-TILs & S-TILs refers to patients with both I-TILs count and S-TILs count exceeding the cut-off value. S-TILs & P-TILs refers to patients with both S-TILs count and P-TILs count exceeding the cut-off value.
